# Epigallocatechin gallate mitigates the motor deficits in a rotenone-induced Parkinson’s disease rat model via promoting protein kinase D1 and inhibiting neuronal Parthanatos

**DOI:** 10.1515/tnsci-2025-0366

**Published:** 2025-02-25

**Authors:** Jianjun Wang, Yaqi Tang, Chenwu Guo, Zekun Du, Fen Chen, Shujuan Fang, Yinjuan Tang

**Affiliations:** Affiliated Hospital, Xiangnan University, Chenzhou, 423000, Hunan, China; Department of Clinical, Xiangnan University, Chenzhou, 423000, Hunan, China; Department of Pharmacy, Xiangnan University, Chenzhou, 423000, Hunan, China; Department of Basic Medical Sciences, Xiangnan University, Chenzhou, 423000, Hunan, China

**Keywords:** Parkinson’s disease, (−)-epigallocatechin-3-gallate, oxidative stress, inflammation

## Abstract

Parkinson’s disease (PD), a neurodegenerative disorder characterized by degeneration of the dopaminergic (DA) neurons, is still lack of available treatments to completely block neurodegeneration. (−)-Epigallocatechin-3-gallate (EGCG), a predominant active polyphenol generated from green tea, exerts multiple neuroprotective roles in the nervous system. However, the function role of EGCG in PD and the underlying mechanism remains to be investigated. In the current study, we used the rotenone injection to build the PD rat model, followed by the EGCG treatment and determined by the behavior tests, measurements of malondialdehyde, glutathione, and superoxide dismutase levels, and enzyme-linked immunosorbent assay. We revealed that, in PD rats, EGCG upregulates protein kinase D1 (PKD1) and inhibits Parthanatos to ameliorate the impaired motor function, reduce the expression of tyrosine hydroxylase, suppress the oxidative stress, and suppress the inflammation in substantia nigra. These combined results suggest that EGCG can suppress oxidative stress and inflammation to prevent DA neuron degeneration to prevent rotenone-induced motor impairments, laying the foundation for EGCG to be a novel candidate for the treatment of PD.

## 1 Introduction

Parkinson’s disease (PD), a kind of chronic neurodegenerative disease featured with degeneration of dopaminergic (DA) neurons [1], is accompanied by multiple clinical motor symptoms, including bradykinesia, rest tremor, and muscular rigidity [2]. Rotenone, a well-known neurotoxin, causes behavioral abnormalities, neurochemical depletion, biochemical alterations, and PD-like neuropathological features, including Lewy body inclusions and α-synuclein aggregation [3], and finally provokes the mechanistic features of PD [4]. It has been previously reported that neuronal cell death induced by rotenone can result in neurodegeneration by inducing inflammation and apoptotic pathways [5,6]. Hence, the plant-derived novel drug candidates with documented antioxidant, anti-inflammatory, and anti-apoptotic properties may be relevant for the prevention of PD [7].

(−)-Epigallocatechin-3-gallate (EGCG) is a predominant active polyphenol isolated from green tea and is considered to be a promising therapeutic candidate for the treatment of diseases associated with oxidative damage and chronic inflammation [8,9,10]. Several experimental studies, including ours, have shown that EGCG can provide neuroprotection against brain, spinal cord injury, sciatic nerve injury, sensory function recovery after dorsal root crush injury, intracranial hemorrhage, and brachial plexus root avulsion [11,12,13,14,15,16].

Protein kinase D1 (PKD1), also called Cµ (PKCµ), is a well-known serine/threonine protein kinase involved in a wide spectrum of functions in both normal states and diseased states [17,18,19]. PKD1 is increasingly associated with a great deal of life processes within the cell [20]. In neurons, PKD1 regulates the exchange of dendritic membrane proteins to exert an essential role in maintaining the polarity of neuron and synaptic plasticity [21]. It has also been indicated that activation of PKD1 is capable of protecting the damaged cells from cell death caused by oxidative insult [22,23,24].

Parthanatos, also called poly(ADP-ribose) polymerase 1 (PARP-1)-dependent death, is a kind of programmed cell necrosis different from necrosis, apoptosis, and autophagy [25] and widely exists in various diseases. PARP-1, as a ribozyme in DNA repair, is considered a risk factor for the Parthanatos progress, has an obvious protective effect in multiple cell insult models, and can improve cell survival status [26]. Hence, Parthanatos is considered to be an essential target for drugs to exert neuroprotective effects.

Given the neuroprotective roles of EGCG in the diseased systems, the neuroprotective effects of EGCG on modulating PKD1 and neuronal Parthanatos in PD remains unknown. Therefore, the aim of the current study is to determine the anti-oxidant and anti-inflammation effects of EGCG on the functional recovery after PD, focusing on its modulating PKD1 and anti-Parthanatos properties.

## 2 Materials and methods

### 2.1 Animals

Male Sprague-Dawley rats with a body weight of 200–220 g purchased from the Guangdong Medical Laboratory Animal Center were maintained in an air-conditioned room on a 12 h light/12 h dark cycle and afforded food and water *ad libitum*.

### 2.2 Rotenone injection and groups

A total number of 50 rats were placed in a stereotaxic apparatus (World Precision Instruments, USA) after being anesthetized with 10% chloral hydrate anesthesia (350 mg/kg). Based on the rat brain atlas to determine bregma, a burr hole was drilled at substantia nigra (SN) anterior-posterior: 5.0 mm, medial-lateral: 2.00 mm, and dorsal-ventral: 8.0 mm [27]. 5 μg/rat at the concentration of 2 mg/kg rotenone was bilateral intracranial injected via the burr hole using a 28-G Hamilton syringe to SN.

To determine the neuroprotective role of EGCG after PD, ten rats per group were randomly divided into five groups: (1) CTRL control, (2) rotenone group, (3) rotenone + EGCG group, (4) rotenone + EGCG + CID755673 (an inhibitor of PKD1) group, and (5) rotenone + EGCG + Ad-PARP-1 (an agonist of Parthanatos) group. The treatment groups were intraperitoneally injected with equivalent phosphate-buffered saline or EGCG (10 mg/kg/day) with or without CID755673/Ad-PARP-1 once daily after injection of 2 mg/kg rotenone for 28 days till sacrifice. The rats without rotenone injection or EGCG treatment were used as CTRL control (*n* = 10/group). All injections were performed at 9:00–11:00 am once daily for 28 days till sacrifice.

### 2.3 Behavioral function tests

All behavioral tests were carried out at 8:00–11:00 am by the experimenters who were blinded to the groups after injection of rotenone and other treatments for 2 h.

#### 2.3.1 Open field test

A plexiglass open field apparatus (72 cm × 72 cm area with 36 cm walls) was used to evaluate the locomotor activity of individual rat [28]. The apparatus floor was divided by lines into 16 squares (18 cm × 18 cm) plus one central square (18 cm × 18 cm). Locomotor activity was indicated by recording the number of squares crossed and the immobility time in 5 min.

#### 2.3.2 Rotarod test

The experiments were conducted as described [29]. All rats were trained on the rotarod apparatus for 3 consecutive days before the injection of rotenone and other treatments. The selected rotor accelerations were 5, 10, 15, and 20 rpm, and the maximum time period of each trial was 5 min. The spent time of rats on the rotating drum was calculated.

#### 2.3.3 Cylinder test

A plexiglass cylinder (30 cm × 20 cm) was used to determine the number of forelimb contacts made by each limb to the apparatus for 5 min.

#### 2.3.4 Catalepsy test

The catalepsy set-up consisted of a vertical grid and a horizontal bar to ascertain inert or static behavior. (1) Grid test [30]: gridiron 30 cm wide and 35 cm high with a space of 1.2 cm between each wire was used. Each rat was hung by all four paws on the vertical grid, and the time taken by each rat to descend the grid was noted as descent latency. Maximum descent latency time was fixed at 180 s. (2) Bar test [31]: rats were placed with both fore paws on a bar, 10 cm above the surface, in half rearing position. The time taken by each rat to remove one paw from the bar was recorded. The maximum latency time was set at 180 s.

#### 2.3.5 Locomotor activity

The locomotor activity was measured by an activity monitor (Opto-Varimex-Mini Model B, Columbus Instruments, Columbus, OH, USA) based on the traditional infrared photocell principle where interruption of 15 infrared beams (wavelength: 875 nm, scan rate: 160 Hz, diameter: 0.32 cm, spacing: 2.65 cm). Rats were habituated in the recording chamber for 2 min, and then, activity was recorded for 5 min and expressed as counts per 5 min [32].

#### 2.3.6 The pole test and traction test

The experiments were conducted as described [33]. In the pole test, a thick rod of 1 cm in diameter and 50 cm in length was first placed in the center of the empty cage, and the time from the top of the rod to the bottom of the cage was recorded for each rat. In the traction test, the ability of the rat to grasp a 0.5-cm-diameter horizontal wire was determined with the following score criteria: 4 points: all limbs can grasp; 3 points: 2 forelimbs and 1 hind limb grip; 2 points: 2 forelimbs grasp; 1 point: only 1 forelimb can grasp; and 0 points: unable to grasp and dropped the wire.

### 2.4 Tissue preparations

After performing all behavioral tests, the SN tissues were collected and homogenized according to previous studies [34] and following centrifugation at 14,000 × *g* at 4°C for 15 min to collect the supernatant for further measurements of malondialdehyde (MDA), glutathione (GSH), superoxide dismutase (SOD), and reactive oxygen species (ROS) levels and enzyme-linked immunosorbent assay (ELISA).

### 2.5 ELISA

The experiments were conducted as described [35,36]. The supernatant was used to determine the protein levels of PKD1 (cat. no. ab131267, Abcam), apoptosis-inducing factor (AIF; cat. no. ab288370, Abcam), PARP-1 (cat. no. ab191217, Abcam), tyrosine hydroxylase (TH; ARD10979, GuideChem), tumor necrosis factor (TNF-α; cat. no. EK0393; WuhanBoster Biological Technology, Ltd.), interleukin-1β (IL-1β; cat. no. EK0412; WuhanBoster Biological Technology, Ltd.), and IL-6 (interleukin-6, cat. no. EK0526; WuhanBoster Biological Technology, Ltd.) using available kits according to the manufacturer’s protocol.

### 2.6 Measurements of MDA, GSH, SOD, and ROS levels

The experiments were conducted as described [37,38]. The commercial assay kits for MDA (cat. no. A003-1-2; Jiancheng Biotech Ltd., Nanjing, China), GSH (cat. no. S0053; Beyotime Institute of Biotechnology, Shanghai, China), SOD (cat. no. A001-3-2; Jiancheng Biotech Ltd., Nanjing, China), and ROS (QC11851, GuideChem, Guangzhou, China) were used according to the instructions [39].

### 2.7 Statistics

All statistical analyses were carried out by GraphPad Prism 6.0 software. Data were reported as mean ± standard deviation and analyzed using one-way analysis of variance followed by the post hoc Bonferroni test. *P < 0.05, **P < 0.01, or ***P < 0.001 indicate statistical significance.


**Ethical approval:** The research related to animals use has been complied with all the relevant national regulations and institutional policies for the care and use of animals. All experimental protocols conducted on rats were approved by The Laboratory Animal Ethics Committee at Xiangnan University.

## 3 Results

### 3.1 EGCG upregulates PKD1 and inhibits Parthanatos in SN of PD rats

To evaluate the effect of EGCG on PKD1 and Parthanatos, ELISA was performed to determine the protein levels of PKD1, apoptosis-inducing factor (AIF), and PARP-1 in SN.

**Figure 1:**
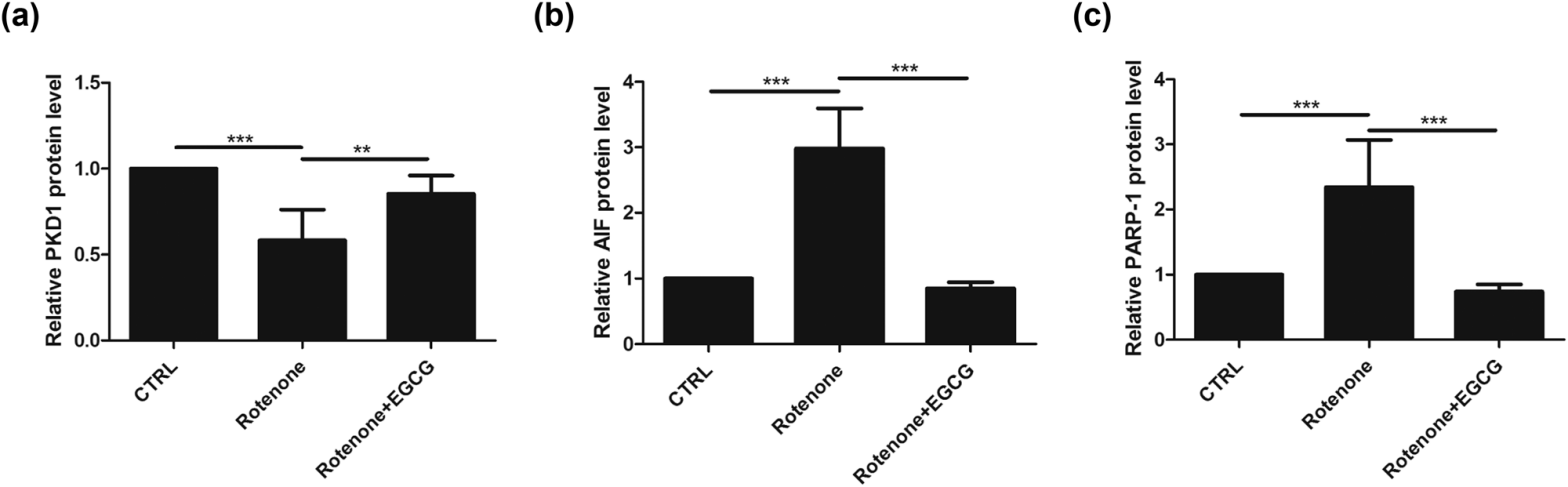
Effect of EGCG on PKD1 and Parthanatos in SN of PD rats. EGCG increased the protein level of (a) PKD1 and inhibited the Parthanatos, as indicated by the decreased protein levels of (b) AIF and (c) PARP-1 (**p < 0.01, ***p < 0.001, *n* = 4).

Compared with the CTRL group, PKD1 level was downregulated in SN of PD rats, whereas EGCG increased PKD1 level (Figure 1a). The reversed patterns for AIF level (Figure 1b) and PARP-1 level (Figure 1c) were also observed.

### 3.2 EGCG upregulates PKD1 and inhibits Parthanatos to ameliorate the impaired motor function in PD rats

To evaluate the effect of EGCG on the motor functional recovery of PD rats, the function assessments (open field test, rotarod test, cylinder test, catalepsy test, locomotor activity, pole test, and traction test) were performed.

**Figure 2:**
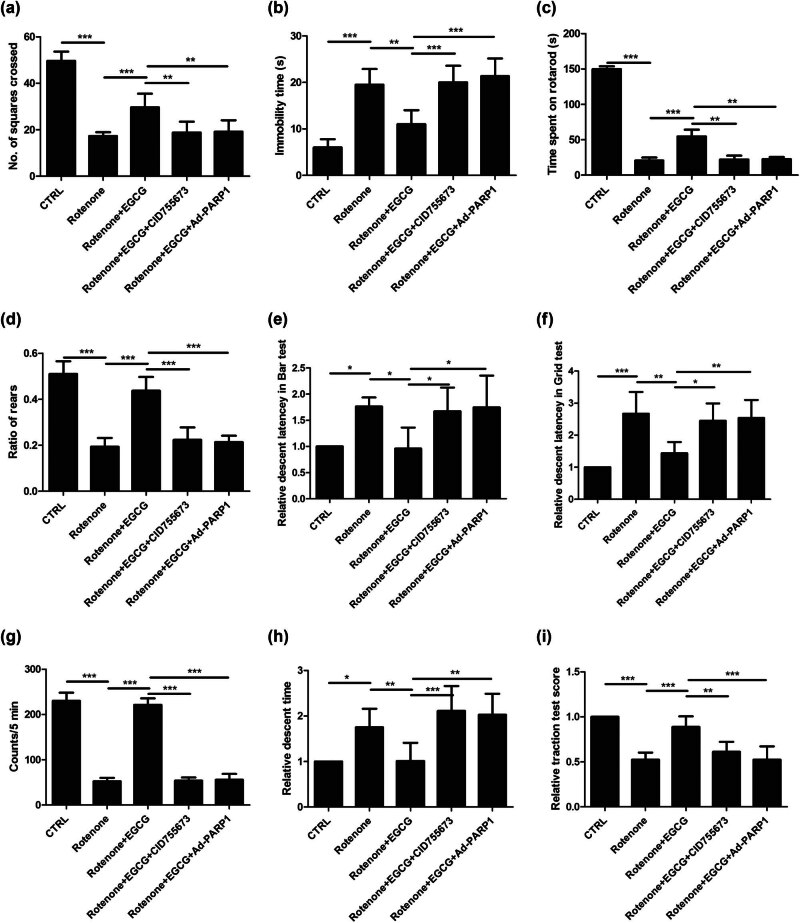
Effect of EGCG on motor impairments in PD rats. EGCG upregulates PKD1 and inhibits Parthanatos to alleviate the motor impairments, as indicated by (a) the increased number of squares of crossed and (b) decreased immobility time in the open field test, (c) the increased time spent on rotarod in the rotarod test, (d) the increased ratio of rears in the cylinder test, (e) the decreased relative descent latency in bar test and (f) the decreased relative descent latency in grid test in the catalepsy test, (g) the increased counts per 5 min in the locomotor activity, (h) the decreased descent time in the pole test, and (i) the increased relative traction test score in the traction test (*p < 0.05, **p < 0.01, ***p < 0.001, *n* = 6).

In the open field test, compared with the CTRL group, the number of squares of crossed was decreased in PD rats, whereas EGCG increased the number of squares of crossed; moreover, EGCG did not decrease the number of squares of crossed in PD rats when CID755673 was used to inhibit the PKD1 expression, and Ad-PARP-1 was used to activate the Parthanatos (Figure 2a). The reverse pattern for immobility time (Figure 2b) to the number of squares of crossed was also observed.

In the rotarod test, compared with the CTRL group, the time spent on the rotarod was decreased in PD rats, whereas EGCG increased the time spent on rotarod; moreover, EGCG did not decrease the time spent on rotarod in PD rats when CID755673 was used to inhibit the PKD1 expression and Ad-PARP-1 was used to activate the Parthanatos (Figure 2c).

In the cylinder test, compared with the CTRL group, the ratio of rears was decreased in PD rats, whereas EGCG increased the ratio of rears; moreover, EGCG did not decrease the ratio of rears in PD rats when CID755673 was used to inhibit the PKD1 expression, and Ad-PARP-1 was used to activate the Parthanatos (Figure 2d).

In the catalepsy test, compared with the CTRL group, the relative descent latency in the Bar test was increased in PD rats, whereas EGCG decreased the relative descent latency in the Bar test; moreover, EGCG did not increase the relative descent latency in Bar test in PD rats when CID755673 was used to inhibit the PKD1 expression and Ad-PARP-1 was used to activate the Parthanatos (Figure 2e). A similar pattern for the relative descent latency in the Grid test (Figure 2f) was observed.

In the locomotor activity test, compared with the CTRL group, the counts per 5 min were decreased in PD rats, whereas EGCG increased the counts per 5 min; moreover, EGCG did not decrease the counts per 5 min in PD rats when CID755673 was used to inhibit the PKD1 expression and Ad-PARP-1 was used to activate the Parthanatos (Figure 2g).

In the pole test, compared with the CTRL group, the descent time was increased in PD rats, whereas EGCG decreased the descent time; moreover, EGCG did not decrease the descent time in PD rats when CID755673 was used to inhibit the PKD1 expression and Ad-PARP-1 was used to activate the Parthanatos (Figure 2h).

In the traction test, compared with the CTRL group, the relative traction test score was decreased in PD rats, whereas EGCG increased the relative traction test score; moreover, EGCG did not increase the relative traction test score in PD rats when CID755673 was used to inhibit the PKD1 expression and Ad-PARP-1 was used to activate the Parthanatos (Figure 2i).

### 3.3 EGCG upregulates PKD1 and inhibits Parthanatos to reduce the expression of TH in SN of PD rats

To further investigate the effect of EGCG on the expression of TH, ELISA was performed to determine the protein levels of TH in SN.

**Figure 3:**
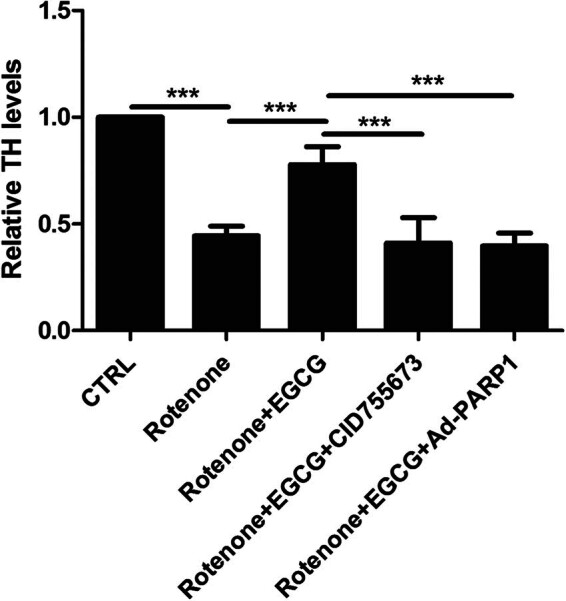
Effect of EGCG on the neuronal survival in SN of PD rats. EGCG upregulates PKD1 and inhibits Parthanatos to increase the expression of TH (***p < 0.0001, *n* = 4).

Compared with the CTRL group, the TH level was downregulated in the SN of PD rats, whereas EGCG increased the TH level; moreover, EGCG did not decrease the TH level in the SN of PD rats when CID755673 was used to inhibit the PKD1 expression and Ad-PARP-1 was used to activate the Parthanatos (Figure 3).

### 3.4 EGCG upregulates PKD1 and inhibits Parthanatos to suppress the oxidative stress in SN of PD rats

To evaluate the effect of EGCG on the oxidative stress in SN of PD rats, measurements of GSH, SOD, MDA, and ROS levels were performed.

**Figure 4:**
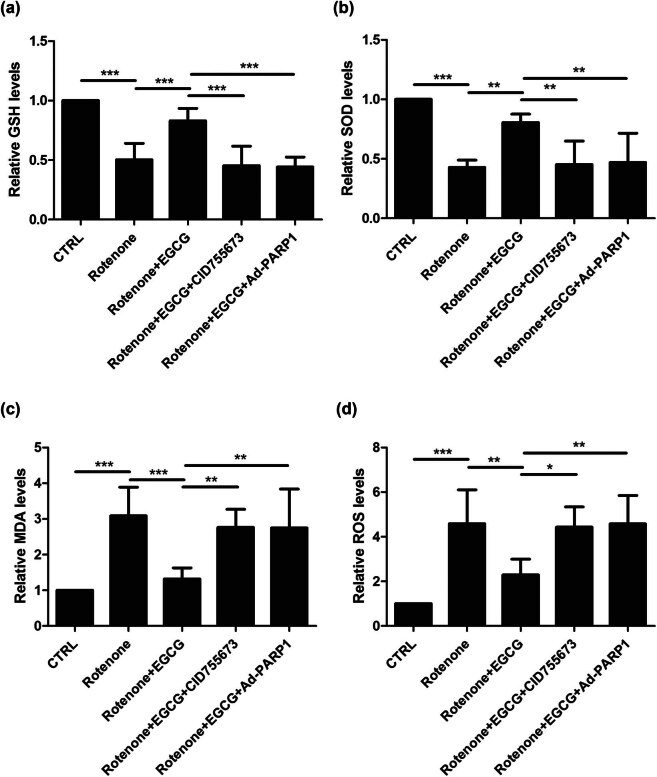
Effect of EGCG on the oxidative stress in SN of PD rats. EGCG upregulates PKD1 and inhibits Parthanatos to increase the levels of (a) GSH and (b) SOD and decrease the levels of (c) MDA and (d) ROS (***p < 0.0001, *n* = 4).

Compared with the CTRL group, the GSH level was downregulated in the SN of PD rats, whereas EGCG increased the GSH level; moreover, EGCG did not decrease the GSH level in the SN of PD rats when CID755673 was used to inhibit the PKD1 expression and Ad-PARP-1 was used to activate the Parthanatos (Figure 4a).

Similar patterns for the SOD level (Figure 4b) and the reverse patterns for MDA level (Figure 4c) and ROS level (Figure 4d) were observed.

### 3.5 EGCG upregulates PKD1 and inhibits Parthanatos to suppress the inflammation in the serum of PD rats

To evaluate the effect of EGCG on the inflammation in SN of PD rats, ELISA was performed to determine the TNF-α, IL-1β, and IL-6 levels.

**Figure 5:**
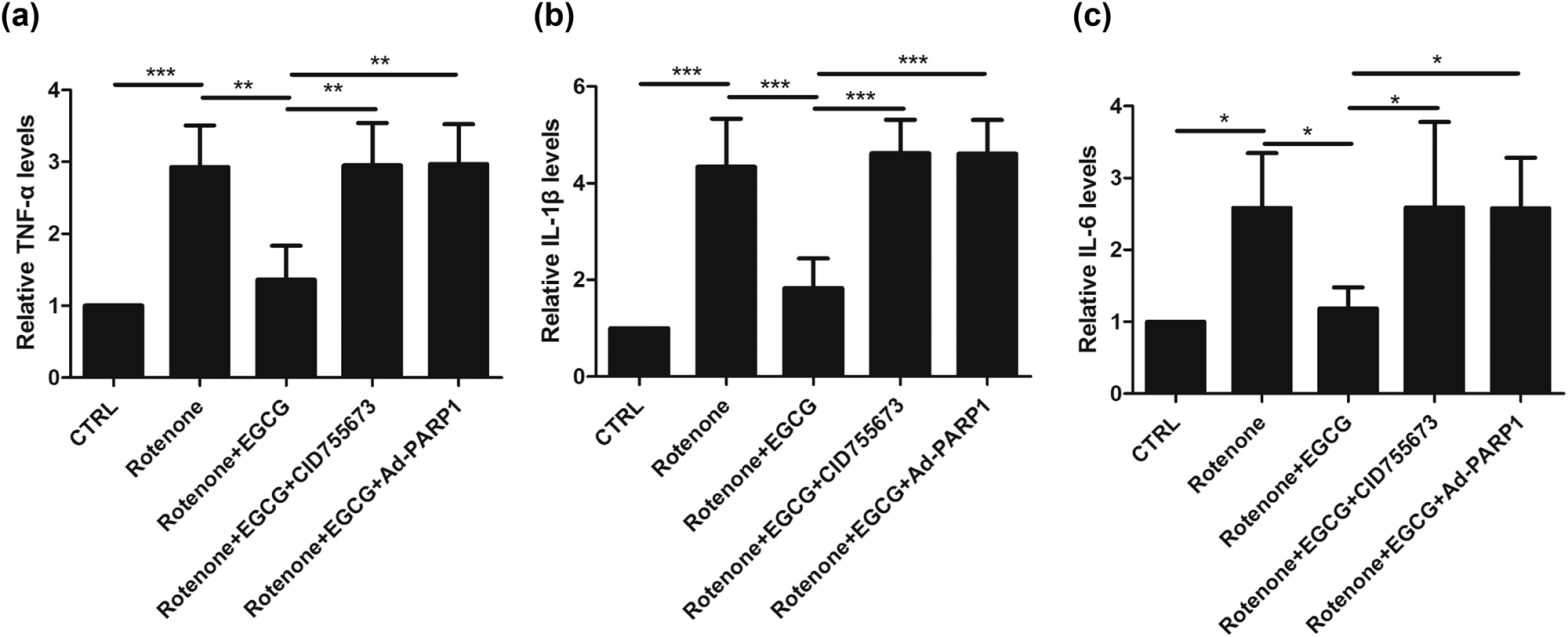
Effect of EGCG on the inflammation in serum of PD rats. EGCG upregulates PKD1 and inhibits Parthanatos to decrease the levels of (a) TNF-α, (b) IL-1β, and (c) IL-6 (*p < 0.05, **p < 0.01, ***p < 0.001, *n* = 4).

Compared with the CTRL group, TNF-α level was upregulated in the SN of PD rats, whereas EGCG decreased TNF-α level; moreover, EGCG did not increase TNF-α level in the SN of PD rats when CID755673 was used to inhibit the PKD1 expression and Ad-PARP-1 was used to activate the Parthanatos (Figure 5a).

Similar patterns for IL-1β (Figure 5b) and IL-6 levels (Figure 5c) were observed.

## 4 Discussion

Substantial efforts have been to identify plant-oriented antioxidant compounds to counteract the mechanisms underlying neurodegenerative disorders like PD [40,41]. In this study, we reported that EGCG can upregulate PKD1 to inhibit Parthanatos to ameliorate the impaired motor function, reduce the expression of TH, suppress the oxidative stress, and suppress the inflammation in SN, suggesting that EGCG may improve the motor impairments via promoting the PKD1 and inhibiting Parthanatos to improve the cell survival of DA neuron in PD.

Assessment of neurological function is a commonly used measure to evaluate the degree of injury and the therapeutic effect of medications [42]. Administration of rotenone in rats can reproduce multiple PD-like behavioral characteristics, including rigidity and hypokinesia [43]. In the present study, we observed that EGCG can ameliorate impaired motor function in PD rats.

Pathological features involved in the development of PD include oxidative stress, misfolded protein accumulation, inflammation, and apoptosis [44]. Oxidative stress may be the key factor leading to Parthanatos [45]. When chemicals in the environment or by-products of oxidative stress damage DNA, PARP-1 is over-activated, causing PAR products to aggregate and further cause nuclear translocation of AIF. Finally, AIF starts nuclear chromatin dissolves or condenses and performs the task of cell death [25]. In the current study, we revealed that EGCG can inhibit the Parthanatos.

Several experimental studies have shown that EGCG can provide neuroprotection against brain, spinal cord injury, and sciatic nerve injury [11,12]. These benefits are mainly due to free radical scavenging or the antioxidant, anti-inflammatory, and anti-apoptotic properties of EGCG [46,47]. EGCG was verified to modulate cell cycle and cell signaling [48] and be against liver injury via its anti-inflammatory and antioxidant effects [49]. Oxidative stress, remarkably increased in the brain tissue of patients with PD [50], plays important roles in the initial degeneration of DA neurons [51]. In the current study, we revealed that EGCG can inhibit the oxidative stress. Chronic neuroinflammation and its elements are accepted as hallmarks of PD progression [52]. Sustained neuroinflammatory products are detrimental to DA neuronal survival [53,54]. In the current study, we revealed that EGCG can inhibit inflammation.

Taken together, treatment with EGCG partially reverses the damage of DA neuron degeneration induced by rotenone to improve motor impairments via promoting PKD1 and inhibiting neuronal Parthanatos, suggesting the effect of EGCG on the treatment of PD.

Although the results are exciting, there are still many limitations, further studies are needed to be performed to detect the effect of EGCG on brain functions, including the striatum, and more methods, including the immunocytochemical staining should be performed, also, the experiments on female animals are needed.
